# Reversible posterior leukoencephalopathy syndrome due to adrenal pheochromocytoma: A case report and literature review

**DOI:** 10.1097/MD.0000000000020918

**Published:** 2020-07-10

**Authors:** Bo Han, Yi Li, Maozhi Tang, Shun Wu, Xiaosong Xu

**Affiliations:** Department of Nephrology, First Affiliated Hospital, Army Medical University (Third Military Medical University), Chongqing, China.

**Keywords:** pheochromocytoma, potential risk factor, reversible posterior leukoencephalopathy syndrome, secondary hypertension

## Abstract

**Rationale:**

Reversible posterior leukoencephalopathy syndrome (RPLS) is a rare neuropathic syndrome with typical clinical and radiological features. There are large amounts of risk factors resulting in RPLS, those including hypertension, eclampsia, neoplasia treatment, renal failure, systemic infections, chemotherapy, and immunosuppressive therapy after organ transplantation.

**Patient Concerns:**

A 27-year-old male patient was admitted for a 2-week history of paroxysmal tic of limbs along with consciousness disorder. Blood pressure elevation was discovered for the first time on admission, and the highest record was 210/150 mmHg during hospitalization. Neurological examinations were positive among mental state, speech, reaction and pathological reflex. The computed tomography scan of the abdomen demonstrated a mass derived from right adrenal gland. The magnetic resonance imaging of the brain showed reversible lesions in the centrum ovale, paraventricular, area and corpus callosum.

**Diagnoses:**

After control of blood pressure and rationally preoperative preparation, the mass was radically resected and verified to be pheochromocytoma by postoperative pathologic findings. He was diagnosed as having RPLS due to adrenal pheochromocytoma.

**Interventions:**

The right adrenal gland mass was completely removed after 2 weeks of α-blockers and β-blockers to treat hypertension.

**Outcomes:**

One week after surgery, the cerebral lesions of RPLS gradually faded and the blood pressure was easy to control well.

**Lessons:**

A few case reports of RPLS related to pheochromocytomas had been documented in the literature. Therefore, we believe that pheochromocytomas may be a potential risk factor of RPLS. If patients receive timely diagnosis and treatment, it can often lead to a favorable prognosis.

## 1 Introduction

Reversible posterior leukoencephalopathy syndrome (RPLS) initially reported by Hinchey et al in 1996 is an uncommon clinical–radiologic syndrome.^[1]^ It is also known as posterior reversible encephalopathy syndrome. Patients with RPLS manifest acute neurological symptoms such as headache, visual disturbances, seizures, encephalopathy.^[2]^ Characteristic radiographic findings of RPLS predominantly include reversible vasogenic edema in the subcortical white and gray matter of the bilateral parietal and occipital lobes.^[3]^ Common pathogenic factors of RPLS include hypertension, eclampsia, neoplasia treatment, renal failure, systemic infections, chemotherapy, and immunosuppressive therapy after organ transplantation.^[4]^

Most of pheochromocytomas are benign tumors, mainly arising from adrenomedullary chromaffin cells, which can produce and release catecholamines, including adrenaline, norepinephrine, and dopamine.^[5]^ Secondary hypertension can occur on account of excess catecholamines and its secretory pattern presenting as continuous or intermittent.^[6]^ If left untreated, it can lead to serious complications involving the cardiovascular system and end organs.^[7]^ Hypertension is an important pathogenic factor of RPLS. In theory, pheochromocytomas may be complicated with RPLS.

## 2 Case presentation

**Figure 1:**
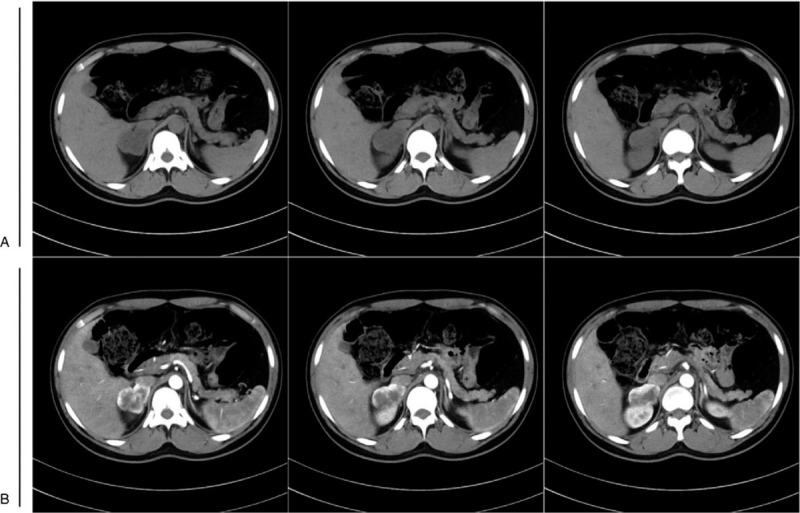
Computed tomography (CT) scan of the right adrenal mass. (A) The mass was showed on non-contrast CT scan. (B) The mass was enhanced in varying degrees on contrast CT scan.

**Figure 2:**
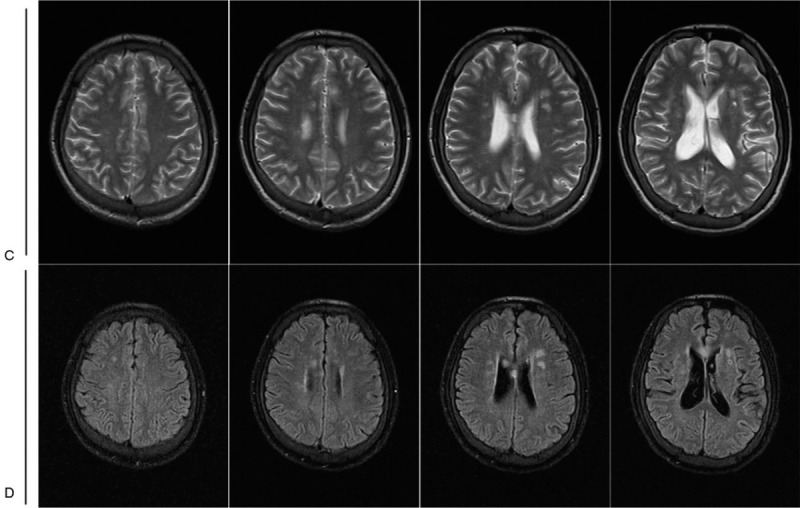
Magnetic resonance imaging (MRI) of the brain. (C, D) Brain MRI showed T2 and FLAIR hyperintensities scattered throughout the white matter of the centrum ovale, paraventricular area and corpus callosum. There was no T2 and FLAIR hyperintensity of the bilateral parietal and occipital lobes.

This 27-year-old male patient was admitted to First Affiliated Hospital of Army Medical University due to a 2-week history of paroxysmal tic of limbs along with consciousness disorder. The symptoms occurred twice and each episode was 2- to 3-minute long. Elevation of blood pressure was firstly found on admission, with the highest recorded blood pressure at 210/150 mmHg during hospitalization. His neurological examinations revealed poor mental state, vague speech, slow reaction, and bradypragia. Bilateral knee reflex presented hyperactivity. Moreover, bilateral patellar clonus, ankle clonus, and babinskin sign could continue to be leaded. There were no sensory deficits and limb muscle strength was normal. Abdominal computed tomography (CT) scan demonstrated an approximate 4.5 cm in diameter mass located in the right retroperitoneum and derived from right adrenal gland (Fig. 1). Brain magnetic resonance imaging (MRI) showed abnormal T2 and fluid-attenuated inversion recovery (FLAIR) hyperintensities scattered throughout the white matter of the centrum ovale, paraventricular area, and corpus callosum (Fig. 2). He exhibited elevated plasma catecholamines, elevated urinary catecholamines, and mildly elevated urinary vanillylmandelic acid. No abnormality was found in blood routine, electrolyte, liver function, and kidney function.

Taking into account the clinical manifestations, the physical examination, the laboratory tests, as well as the imaging changes, preliminary diagnosis of the patient was adrenal gland mass with hypertension and RPLS. After 2 weeks of α-blockers and β-blockers to treat hypertension, the adrenal gland mass was completely removed and intraoperative blood pressure was relatively stable. Postoperative pathologic findings verified that the mass was pheochromocytoma. One week after surgery, the patient's blood pressure controlled well without medication and cerebral lesions gradually subsided. There is no evidence of recurrence after 1 year of follow-up. At the present time, the patient is completely asymptomatic.

## 3 Discussion

In large part, thanks to improved and more readily available brain imaging in recent years, RPLS is becoming increasingly recognized by clinicians.^[2]^ Because of massive causes, diverse clinical manifestations, and complicated imaging features, RPLS is especially easy to be misdiagnosed. Although the syndrome is generally reversible both clinically and radiographically, a delay in effective therapy also may induce severe and irreversible neuronal cell death.^[8]^ To date, the pathophysiological mechanism of RPLS is still not clear. The hypotheses of cerebral blood flow autoregulation failure and endothelial dysfunction are leading theories. If the cerebral blood flow autoregulation fails because of abrupt and severe blood pressure elevation exceeding the upper limit of itself, hyperperfusion can occur and induce breakdown of the blood–brain barrier, then plasma and macromolecules infiltrates into the interstitium of brain cells and vasogenic edema happen in the end.^[9]^ In addition, endothelial injury caused by direct effects of excessive cytokines in the circulation can increase vascular permeability, which also gives rise to vasogenic edema subsequently.^[10]^

A definitive diagnosis of RPLS largely depends on the typical imaging features. Although the lesions of RPLS can be detected by both CT and MRI technologies, MRI has more advantages in finding early lesions and small local abnormalities due to the higher sensitivity to soft tissue edema than CT. Thus, MRI is the most important tool of observing RPLS lesions.^[11]^ Brain MRI of RPLS demonstrates bilateral asymmetric T1 hypointensities or isointensities, T2 and FLAIR hyperintensities commonly scattered throughout the parietal and occipital regions, occasionally in other areas such as the cerebellum, brain stem, basal ganglia, and the spinal cord.^[12–13]^ Moreover, our report shows that the centrum ovale, paraventricular area, and corpus callosum can also be involved.

A few case reports in the literature indicated that pheochromocytomas can lead to occurrence of RPLS (Table 1).^[14–16]^ Large amounts of catecholamines are continuously or intermittently released by tumor cells, which can bring about secondary elevation of blood pressure with wide amplitude of fluctuation. A sudden change in blood pressure is likely to result in RPLS. Therefore, pheochromocytomas may be a potential risk factor of RPLS. It should not be ignored during clinical diagnosis and treatment.

**Table 1:**
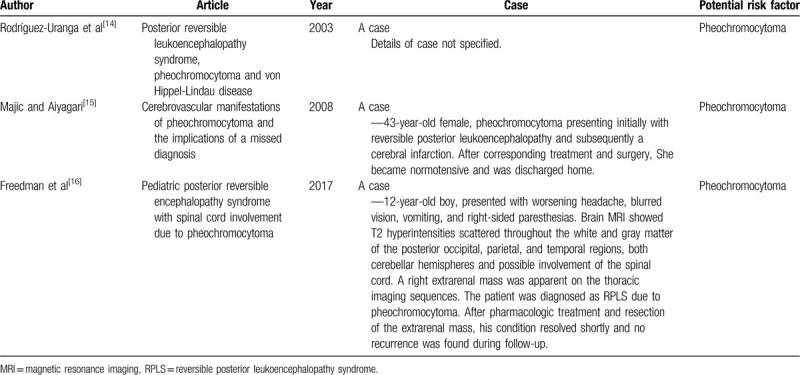
Cases of RPLS due to pheochromocytoma.

The patient with seizures and secondary hypertension had typically radiographic evidences of RPLS, and his primary cause was the right adrenal pheochromocytoma. After symptom-pointed treatment and radical resection of adrenal lesions, the condition of the patient gradually improved and the subsequent follow-up showed complete recovery. In conclusion, clinicians should pay more attention to pheochromocytomas as a potential cause of RPLS. Accurate diagnosis and effective treatment can often lead to a favorable prognosis.

## 4 Patient consent

The patient had provided informed consent for the writing and publication of this case report.

Written informed consent had been obtained from the patient for the publication of this case report and any accompanying images.

## Author contributions

**Conceptualization:** Bo han, Xiaosong Xu.

**Formal analysis:** Bo han, Yi Li, Maozhi Tang, Shun Wu, Xiaosong Xu.

**Investigation:** Bo han, Yi Li, Shun Wu, Xiaosong Xu.

**Methodology:** Bo han, Yi Li, Maozhi Tang, Xiaosong Xu.

**Project administration:** Yi Li, Maozhi Tang, Shun Wu, Xiaosong Xu.

**Resources:** Bo han, Maozhi Tang, Shun Wu, Xiaosong Xu.

**Writing – original draft:** Bo han.

**Writing – review & editing:** Yi Li, Maozhi Tang, Shun Wu, Xiaosong Xu.

## References

[1] Hinchey J Chaves C Appignani B A reversible posterior leukoencephalopathy syndrome. N Engl J Med 1996;334:494–500. 8559202 10.1056/NEJM199602223340803

[2] Fugate JE Rabinstein AA Posterior reversible encephalopathy syndrome:clinical and radiological manifestations, pathophysiology, and outstanding questions. Lancet Neurol 2015;14:914–25. 26184985 10.1016/S1474-4422(15)00111-8

[3] Gao B Yu BX Li RS Cytotoxic edema in posterior reversible encephalopathy syndrome:correlation of MRI features with serum albumin levels. Am J Neuroradiol 2015;36:1884–9. 26138140 10.3174/ajnr.A4379 PMC7965038

[4] Marrone LCP Gadonski G Diogo LP Posterior reversible encephalopathy syndrome:differences between pregnant and non-pregnant patients. Neurol Int 2014;6:5376. 24744848 10.4081/ni.2014.5376 PMC3980148

[5] Melkozerov KV Kuznetsov AB Kalashnikov VY Sustained ventricular tachycardia in a patient with a single ventricle of the heart and a pheochromocytoma. Probl Endokrinol (Mosk) 2019;65:107–12. 31271713 10.14341/probl9949

[6] Canu L Parenti G De Filpo G Pheochromocytomas and paragangliomas as causes of endocrine hypertension. Front Endocrinol 2019;10:333. 10.3389/fendo.2019.00333 PMC6558199 31214117

[7] Siddiqui MA Mittal PK Little BP Secondary hypertension and complications: diagnosis and role of imaging. Radiographics 2019;39:1036–55. 31173541 10.1148/rg.2019180184

[8] Shen T Chen H Jing J A study on clinical characteristics and the causes of missed diagnosis of reversible posterior leukoencephalopathy syndrome in eclampsia. Neurol Sci 2019;40:1873–6. 31062190 10.1007/s10072-019-03914-3

[9] Cruz RJ Jr DiMartini A Akhavanheidari M Posterior reversible encephalopathy syndrome in liver transplant patients:clinical presentation, risk factors and initial management. Am J Transplant 2012;12:2228–36. 22494636 10.1111/j.1600-6143.2012.04048.x

[10] Marra A Vargas M Striano P Posterior reversible encephalopathy syndrome:the endothelial hypotheses. Med Hypotheses 2014;82:619–22. 24613735 10.1016/j.mehy.2014.02.022

[11] Chen S Hu J Xu L Posterior reversible encephalopathy syndrome after transplantation:a review. Mol Neurobiol 2016;53:6897–909. 26666662 10.1007/s12035-015-9560-0

[12] Fischer M Schmutzhard E Posterior reversible encephalopathy syndrome. J Neurol 2017;264:1608–16. 28054130 10.1007/s00415-016-8377-8 PMC5533845

[13] McKinney AM Short J Truwit CL Posterior reversible encephalopathy syndrome: incidence of atypical regions of involvement and imaging findings. Am J Roentgenol 2007;189:904–12. 17885064 10.2214/AJR.07.2024

[14] Rodriguez-Uranga JJ Franco-Macias E Bernal MSA Posterior reversible leukoencephalopathy syndrome, pheochromocytoma and von Hippel-Lindau disease. Rev Neurol 2003;37:797–8. 14593644

[15] Majic T Aiyagari V Cerebrovascular manifestations of pheochromocytoma and the implications of a missed diagnosis. Neurocrit Care 2008;9:378–81. 18509763 10.1007/s12028-008-9105-8

[16] Freedman D Koram A Gillson N Pediatric posterior reversible encephalopathy syndrome (PRES) with spinal cord involvement due to pheochromocytoma. Pediatr Neurol 2017;77:92–3. 28939016 10.1016/j.pediatrneurol.2017.06.016

